# Experiences of living with postural tachycardia syndrome

**DOI:** 10.1177/17423953211054032

**Published:** 2021-11-17

**Authors:** Samantha Waterman, Morwenna Opie, Debbie Waterman, Dawn Langdon

**Affiliations:** 1 Department of Psychology, 3162 Royal Holloway University of London, Egham, UK; 2 Clinical Psychologist, 40384 Duchy Hospital, Truro, & Trustee, PoTS UK, Truro, UK; 3 1174 Origins Insights Healthcare Communications, Fleet, UK

**Keywords:** Postural tachycardia syndrome, autonomic dysfunction, dysautonomia, qualitative analysis, chronic illness

## Abstract

**Objective:**

Postural tachycardia syndrome (PoTS) is a disorder of the autonomic nervous system which involves a range of symptoms, worsened when adopting an orthostatic (upright) position. Symptoms can include tachycardia, dizziness, fainting, nausea as well as many others which, as is typical of a syndrome, vary from person to person. Although research is increasing into this condition, the unifying experiences of managing it on a daily basis have not been extensively investigated. This study aimed to capture participants’ experiences of living with PoTS.

**Method:**

A longitudinal digital ethnographic approach was employed. Eight participants recorded daily video diaries discussing their experiences of PoTS and its impact for 17 days. Interpretative phenomenological analysis was utilised to analyse the data and identify connections across participants’ accounts.

**Results:**

Four superordinate themes emerged: ‘loss of control and lack of agency over body’, ‘identity changes’, ‘lack of understanding from others’ and ‘adapting to cope with PoTS’.

**Discussion:**

The findings demonstrated the complex and widespread impact these participants experience from their PoTS symptoms, including the consequent emotional difficulties that result from managing this condition. An overall lack of understanding about PoTS by others was emphasised, suggesting the requirement for better education and support services for this condition.

## Introduction

Postural tachycardia syndrome (PoTS) is a chronic health condition associated with dysfunction of the autonomic nervous system.^1^ Studies suggest the prevalence range in developed countries is between 0.2% and 1.5% of the population.^2,3^ PoTS has a significant female predominance with a female-to-male ratio of 5:1^4^ and is more common in people under the age of 40 years.^5^

PoTS is a heterogeneous disorder and causes a constellation of symptoms that vary significantly from person to person and have a variety of triggers.^4,6^ As the dysfunction relates to the autonomic nervous system, the condition generally has a multisystem impact.^7^ Due to the diversity in presentation, as well as lack of clarity around the underlying mechanisms, diagnosis can be delayed by several years^8^ and many patients are misdiagnosed as experiencing panic disorder or other psychiatric conditions.^9^

Once a diagnosis is made, the debilitating nature of PoTS can have a significant impact on a person’s quality of life (QoL).^6,10^ Due to the fluctuating nature of the condition, people with PoTS can often appear well for periods of time despite being severely incapacitated more generally.^11^ Although the condition is not associated with an increase in mortality, functional disability is reportedly similar to that found in chronic obstructive pulmonary disease and congestive heart failure.^12^

While previous large questionnaire-based studies in this group have detailed the symptoms people with PoTS experience, very little has been described about the intricacies and ongoing impact of these on daily life. Pharmacological treatment of PoTS does not generally result in a complete resolution of symptoms,^8^ thus, it is crucial to gain an in-depth understanding of the functional and psychological impact of PoTS to develop the most effective interventions. While QoL questionnaires can be used, they only capture a snapshot of a person’s experience and there are no specific validated PoTS QoL scales. Using a longitudinal qualitative approach, this study aimed to look at the fluctuations in symptoms over time and gain a detailed insight into the impact of this to help further our understanding of PoTS and to develop better interventions for this population.

## Method

### Design

Interpretative phenomenological analysis (IPA) using a longitudinal qualitative design was deemed the most suitable approach. IPA is an inductive approach that specifically aims to investigate an individual's experiences and how they make sense of this themselves.^13^ IPA is particularly well suited to studies exploring healthcare issues,^14^ as well as their impact on participants’ lives.^15^ Focused digital ethnography, with an unstructured approach to interviewing^16^ was used as the method of data collection. In this study, data were gathered using daily video diaries recorded remotely by participants. IPA has been noted as a complimentary analysis method when utilising an ethnographic approach as it offers a more holistic output, including both descriptive and interpretive commentary on the data.^17^

### Ethics and risk

Ethical approval was obtained from the Research Ethics Committee of the Royal Holloway University of London and appropriate information sheets and consent forms were administered in line with this. Each participant had an opportunity to ask questions and then gave informed consent to participate. Post-enrolment participant remuneration was sourced; the reimbursement was not mentioned in the advert so as not to unduly influence people’s choice to participate.^18^ A risk protocol was in place and all videos were reviewed by a trainee clinical psychologist within 24 h of uploading to the platform to help manage any emotional distress. A clinical psychologist was on hand to support participants if needed, however, this was not required during the study.

### Recruitment

Participants were recruited through an advertisement published on the PoTS UK charity Facebook page and through their email newsletter. The inclusion criteria were English-speaking people over the age of 16 years who had been diagnosed with PoTS by a medical professional and who had experienced symptoms for at least 6 months. Exclusion criteria included severe cognitive impairment and/or lacking the capacity to consent to participate in the study or currently an inpatient for either their psychological or physical health care needs. Purposive sampling was employed to ensure at least one male was recruited.

### Procedure

**Figure 1.**
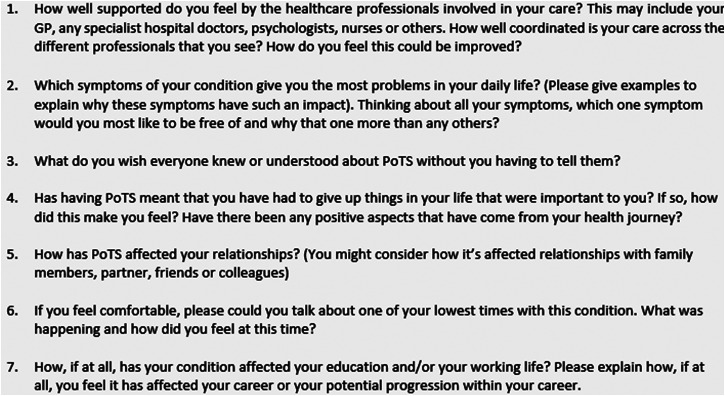
The seven ‘secret questions’.

Once recruited participants received a kit that contained a mobile phone tripod, seven sealed envelopes containing ‘secret questions’ and a user guide for the mobile application where they uploaded their videos. Participants were asked to record for 17 days; this time period has been used in a previous study and shown to be effective in capturing change over time.^19^ Participants were asked to record for approximately 10 min per day and discuss their experience of PoTS that day. On seven of these days, participants were asked to open one of the ‘secret question’ envelopes and answer the question on camera as part of their recording for that day (Figure 1). These questions provided a basis for discussing topics that would help answer the research questions posed by the study and helped to give participants a focus and promote engagement. The questions were collaboratively generated with input from the PoTS UK charity.

Recording took place between 2 March 2020 and 5 April 2020. Participants were encouraged to record consecutively for the length of the project, although seven participants had to take a break due to feeling too unwell to film as a result of PoTS symptoms, or difficulties relating to the COVID-19 pandemic as the UK lockdown started on 23 March 2020.

### Analysis

The full 17-day transcript for each participant was analysed, using NVIVO for data management, following an IPA approach.^20^ Transcripts are analysed one person at a time, data are read and goes through three layers of theme development: exploratory notes, emergent themes, connecting emergent themes. Once all individual transcripts were analysed, a cross-case analysis was completed to highlight convergences and divergences across cases, and the final themes were drawn out to create an overall representation of participant experience.

The data collection coincided with the start of the COVID-19 pandemic and, while illuminating and useful for considering future policy, themes associated with the pandemic have been excluded from this current analysis and will be disseminated separately as they were very specific to this extraordinary time period.

Following analysis, respondent validation was utilised in an effort to increase the credibility of the analysis and ensure affinity between the researcher and participants’ understanding of their experience.^21^ This was deemed important due to the ethical implications of interpretation potentially imposing meaning on a person’s experience that may not appropriately fit.^22^ Participants were emailed a full copy of the results and interpretation and asked to share any comments. All participants replied and were unanimously happy with the results, with many noting it was helpful to hear others experiences, therefore, no revisions were required.

## Results and discussion

### Participants

The final sample consisted of eight people aged 20–47 (mean age: 29.37), seven female and one male. Seven participants identified as ‘White British’ and one identified as ‘Mixed White/Middle Eastern’. Time since diagnosis ranged from 2 weeks to nine and a half years, but all had been experiencing symptoms for more than a year. All participants completed the study and total length of individual’s recordings ranged from 1 h 43 min to 7 h 45 min.

### Loss of control and lack of agency over body

An undercurrent to all narratives was a sense of losing control and agency over their body as a result of this condition. The breadth and intensity of symptoms experienced by participants over the relatively short filming time demonstrated the widespread impact this condition has on the body. Typical examples include this extract where participant 6 struggles with brain fog. Although these symptoms are often considered invisible, they are more tangibly demonstrated here:

‘*I can't, like, with PoTS, again, when you‘re tired you can't filter noise, so I’m hearing talking, and I can't focus on what I’m saying. But yeah, so it meant that [frowning] lost my train of thought. Ooh, my train of thought’ (P6)*

All participants talked about areas of their lives where restrictions had been imposed by physical symptoms outside of their control. The struggle articulated by many participants between their ‘self’ remaining the same but their body being restricted by their symptoms is encapsulated here:

‘*I remember thinking I just don't want this body. I just want to unzip this body that makes me feel so tired and get in someone else's body who's jogging past me. And I just wanted to run. … just to run and run and run and run and wherever I got to when I ran, I just hoped that I’d feel better then.’ (P6)*

The majority of participants also reflected on the reciprocal relationship between their symptoms and their emotions. A repeating theme was the conflicting response caused by physical symptoms which mimic the experience of anxiety (e.g. tachycardia), which were reportedly hard to reconcile and understand.

‘*I’ll go to bed, there’ll be nothing that actually physically worries me…but my heart, I lie down my heart will be racing and I‘ll be short of breath and I won't feel anxious but it causes that I feel that, and then in my head I’m like I feel really like, scared, I don't know what I’m scared of, and I have to tell myself like It's just PoTS, but it, ‘cause it's a body response it's really hard’ (P6)*

Participant 6 speaks of cognitively feeling calm and content but physically feeling very on edge. This was a common theme in other transcripts and, as illustrated here, the dissonance between a conscious knowing that sensations are PoTS associated, and the inevitable impact on feelings of safety and well-being from this uncontrollable feeling of distress and physiological arousal is hard to resolve.

The fluctuating nature of PoTS and the substantial impact of this unpredictability were addressed by all participants. This has a wider impact on relationships as well as on education and work, which supports previous quantitative research in this population.^8,9^ This aspect of PoTS seemed to be one of the most debilitating; participants felt they were a slave to the condition due to the wide range of triggers as well as symptoms often flaring with no warning:

‘*One day you can be absolutely, not absolutely fine but you can be relatively okay and the next day, you could be really unwell, and fainting numerous times throughout the day’ (P8)*

*‘It's not just standing up that can trigger. Exercise could trigger, and foods, and drink. Not enough food, not enough drink. Particular foods, particular drinks. Being too hot. If somebody's being too cold and then getting too hot quite suddenly, or the other way round. Being in quite a dry environment. Tiredness, stress.’ (P2)*

Interestingly, there were many occasions where participants attempted to unpick why their symptoms may have increased, perhaps as a way to regain a sense of control in the face of constant unpredictability. For some, it appeared that there was also an emotional toll from this process and the blame they assign to themselves when their condition flares.

‘*So, it was a bit frustrating [symptom flare]. I probably got my food intake a bit wrong yesterday. I probably had too much dinner. Also, I think I’ve probably had a little bit too much dairy recently.’ (P4)*

*‘…sometimes, you know, you still make, not mistakes, I don*‘*t want to say mistakes, but it can feel like, you feel like you should know what to do to make yourself well. And some days you just still feel ill, and you‘re like, “I‘m trying my best” (P6)*

Conversely, there were also many times when participants would show a resignation and acceptance that this is just another part of their condition:

‘*So, it has been a pretty bad day today, but, unfortunately, that is just one of the things that you have to try and overcome when living with PoTS, some days are worse than others’ (P5)*

It was not always clear whether this was a productive coping mechanism or possibly more an exhausted acquiescence of control to the symptoms.

### Lack of understanding from others

Participants unanimously expressed a lack of understanding from others about PoTS and people not always appreciating their needs, which compounded the difficulties of living with the condition. Participants often described how others underestimated the struggle they experienced and the daily impact the condition has:

‘*The headaches that come with it, the coat hanger pain, it is just relentless symptoms and I don't think people understand how awful it is to live with on a day-to-day basis.’ (P1)*

*‘it's harder to live with than it looks! Many people assume just because you have PoTS, it only affects you when you stand up because your heart rate goes up, but it's so, so much more than that, like, every day is a struggle.’ (P5)*

Additionally, participants voiced that this lack of understanding is likely to be exacerbated by the invisible, indescribable and immeasurable nature of many of the symptoms, making it more difficult for others to comprehend. This difficulty to explain or share subjective experience is known to compound the emotional impact of not being understood (Pinel et al. 2015), exacerbating feelings of isolation and loneliness which can have a significant impact on health outcomes.

‘*I mean I’ve got a lot of understanding people around me, but I can tell they don't know what I‘m talking about. Especially even things like yesterday with the fatigue, even joint pain, things like that are very difficult to explain to people.’ (P7)*

There was a sense across all transcripts of the emotional toll that results from constantly feeling misunderstood, with many participants using epizeuxis to emphasise their acute levels of distress:

‘*I want people to understand that even though I look well… I really really am not feeling well.’ (P3)*

As well as a lack of understanding, many participants shared experiences of being negatively judged by others. Often this is closely related to the invisible nature of the condition not matching up with people’s expectations of someone with a disability.

‘*The amount of times I’ve had the glare that I put my blue badge up and somebody says is that for you?.’ (P8)*

*‘there's often about 8 flights of stairs so I’m just there waiting for the lift while everyone else goes upstairs… and people kinda look at me as if to say like “Why are you taking the lift? You’re a young person, you should be walking."’ (P5)*

This aligns with previous research where participants have expressed that the invisible nature of PoTS can be both a ‘blessing and a curse’. Although it can sometimes reduce the overt discrimination, it can equally result in judgement of needing ‘unnecessary’ support, because people with PoTS often appear ‘able-bodied’.^23^ More broadly this builds on research investigating other chronic conditions with unseen symptoms, such as chronic fatigue syndrome,^24^ fibromyalgia^25^ and more recently ‘long-COVID’^26^ where feelings of invisibility and stigma due to others not understanding the condition are prevalent. This can lead to people feeling more isolated, impacting negatively on their response to their diagnosis as well as their attempts to seek support, compounding this isolation.^27^

Mental well-being was explored by all participants, with some candidly discussing the direct impact of PoTS and reduced functioning on their mental health; describing the isolation and in some cases depression that they have experienced. Although no participants in this study reported suicidal ideation, more than one voiced that they felt like a burden on those around them at times, particularly due to the fluctuations in their condition which meant they could unexpectedly need support at short notice:

‘*I feel like I’m helping inadequately if that makes sense. I felt like I was a burden’ (P1)*

*‘I’ll get very upset and overtired and I won't understand what he's saying and then I feel upset that I don't understand what he's saying and sometimes I can feel like a burden’ (P6)*

The psychological impact of living with a chronic illness should not be underestimated; rates of suicidal ideation and completion are particularly high in chronic invisible illness groups. In previous studies, more than 50% of the PoTS participants have been shown to be at high risk for suicide and the feeling of being a burden to others is the strongest predictor of suicidal risk in this group.^28,29^ This may be explained by the interpersonal theory of suicide, which posits increased burdensomeness as a key factor for increasing suicide risk.^30^ This is important for professionals supporting this client group to consider as it may predict future suicide risk.

Finally, almost all participants shared experiences of healthcare professionals (HCP) not understanding PoTS and consequently not always receiving the appropriate care. The emotional impact of feeling dismissed was reflected in the following extracts:

‘*I was constantly being dismissed…. The doctors that I had at the time had no idea what was wrong with me and put it all down to anxiety.’ (P1)*

*‘See, I’ve very much felt like, even though people [HCPs] have been interested in me, I’ve been a bit like a parcel. (Laughs) It's taken a very long time to be delivered anywhere’ (P3)*

Nearly all participants wanted greater understanding from HCPs that QoL is as important as survival, and PoTS should not be underestimated just because it is not life threatening, as illustrated below:

‘*…that's part of what makes your life rich, and that's part of what makes your life an enjoyable life rather than just like an existence…There's more to life than just staying alive. And I think that this assumption that I think healthcare settings can have of if you‘re not likely to die imminently then you‘re kind of okay is so unhelpful because there's so much more to life than that.’ (P2)*

The many experiences of others not understanding their condition becomes compounded when they reach out to HCPs and experience the same lack of awareness. Nearly all participants reported experiencing a lack of knowledge and understanding from HCPs across primary and secondary care. It was less clear whether this same experience occurred in tertiary services, although it is of note that specialist tertiary services are few and far between for PoTS. Given the difficulties most participants noted towards the start of their journey with PoTS, it is perhaps understandable that there is often distrust in doctors amongst this community. The positive flip side of this appears to be that when they do develop good relationships with their medical teams, this is a rewarding experience for all parties. Positive experiences with HCPs were often due to the knowledge, interest or efforts of the professional in learning about PoTS, which then improved subsequent interactions.

‘*One doctor in particular is really good and he was like ‘bring any information I’m always really inquisitive‘ and he knew a bit about it anyway and that was really nice’ (P6)*

*‘I tell my team often how grateful I am for them and how lucky that I am to sort of have them on my side. And they know me really well, …. And we‘ve built up an incredible relationship, which I‘m so, so thankful for.’ (P1)*

These comments demonstrate the value of being heard and understood, even if it is HCPs admitting they need to learn more about the condition. Since participants have described many situations where others have not understood their needs, having someone offer to learn more about it to better support them is likely to be an incredibly validating, potentially therapeutic, experience.^31^

### Identity changes

Overall participants reported a significant impact to their identity and sense of self since developing PoTS, although their experiences and reactions varied. A number of participants spoke about feeling different from others since the onset of PoTS, often comparing their physical limitations now to the freedoms of their peers. There was a sense in some narratives of losing their past selves and in some, no longer feeling ‘normal’:

‘*we were sat down for about two and a half hours without a break so obviously for a normal person even being sat down that long it*‘*s going to be weird when you stand up so for me it was double weird’ (P5)*

*‘I think, sometimes dealing with the…* *mental health of a physical condition, means that you do separate yourself, you see yourself as alien or you see yourself as different’ (P6)*

Both participants seem to have ostracised themselves from their concept of ‘normal’; while this may be a useful coping strategy to their sense of difference, it may also be isolating and detrimental over time. It was not clear in the narratives whether this shift in identity was self-perceived, possibly based on the gap between their expected self and their perceived self, or whether this related to experiences from those around them. Given the theme around lack of understanding from others being so pervasive throughout the videos, the latter may be more likely. This is similar to findings from a recent qualitative study investigating the impact of rheumatoid arthritis, where a shift in identity was a central theme in participant’s lives.^32^ In this case, the shift was purported to relate to being treated differently by others and thus the client coming to no longer recognise themselves, developing a poor body self-unity. It is possible a similar process occurs in PoTS patients, exacerbated by others not understanding their experience.

Conversely, some participants expressed that they had experienced a lot of change due to their condition but actually now considered PoTS an integral part of who they were, a ‘new normal’. In some, this shift in identity and self-concept seemed to come out of necessity to survive, with this approach serving as a protective mechanism helping them carry on with their life.

‘*I very much was like, okay I’ll just accept this, and take it with me as I move on with my life. I think if I were to have been really bothered, by the idea of being ill, having PoTS. I think that would have really stopped me, from getting on with my life.’ (P2)*

### Adapting to cope with PoTS

Most participants discussed the multitude of ways they had adapted aspects of their worlds to cope better with PoTS and work around their symptoms. Nutrition was a common coping strategy that was noted by many participants. Dietary changes such as increasing sodium intake,^33^ reducing refined carbohydrates and foods high in fat and fibre are often recommended in this population who are more likely to struggle with delayed gastric emptying.^34^ Although there is further research needed into dietary recommendations, and which strategies worked best differed amongst participants, likewise with exercise for those who were able to tolerate it:

*‘I have tried like a low carbohydrate diet, which tends to work for me best, low carb to zero carb. I’ve tried high fat…I’ve tried like a meat and vegetables type diet, like a ketogenic diet. I’ve tried a few’ (P7)*

*‘I do find that doing exercise seems to keep my strength up and adds to the amount that I can walk without feeling like I’m going to crash.’ (P4)*

A plethora of unique coping strategies employed by participants was mentioned from service dogs which can detect heart rate changes to breakfast bars that enable seated cooking, reflecting the need for creativity in developing skills to manage this condition. It would seem from the participant’s entries that it is important not only to prevent symptom escalation, but also to attempt to regain the control they feel they have lost at the hands of PoTS and reclaim a sense of agency over their well-being. It may equally reflect the scarcity of available treatment for this condition. Additionally, due to the variation in symptoms and the multisystem involvement, it is likely that people with PoTS will be under the care of multiple specialties, but it was clear from these participants that having a supportive and connected medical team was a validating and empowering experience for them. Complex disability such as this requires an integrated multidisciplinary approach; the benefit of PoTS specialist teams should be acknowledged.

### Strengths and limitations

A key strength of this study is that it is the first longitudinal qualitative study in this population, which has enabled a clearer understanding of the day-to-day fluctuations and impact of this condition. By utilising a remote digital approach to data collection, participants were able to record flexibly which supported participant retention as they could fit their recording around symptom flares and still remain engaged with the study.

Although the findings from this study reflect a particularly homogenous sample, this is similar to PoTS samples from large cohort studies in this population. PoTS has a significant female predominance and the current largest scale study looking at the characteristics of people with PoTS found there was a significant over-representation (93%) of White participants.^9^ This pattern has also been found in paediatric PoTS samples,^35^ suggesting this sample may be representative of the wider PoTS population. However, there may be groups that are unable to access a diagnosis and thus are not represented in this or other studies to date. Finally, the study was co-designed by someone with PoTS (SW) and with the support of the PoTS UK charity, which enabled an expert-by-experience perspective to influence the whole project. Participants engaged in the study were aware that the research was linked with the PoTS UK charity and a member of the research team had PoTS. It was noted by many that this made them feel more comfortable sharing the more difficult aspects of the condition, as they felt they would be understood.

However, the study also has a number of limitations. While IPA findings are often not suitable for generalisation to wider populations due to their idiographic focus, there is likely a recruitment bias due to the sample being self-selecting through the PoTS UK charity. It is possible that people who actively engage with the PoTS UK charity may have more difficulties managing symptoms and their condition has a more significant impact on their lives. Additionally, due to the recruitment method and the study being digitally based, it is likely the participants represent a younger, affluent and technologically capable group. It would be beneficial for future studies to undertake qualitative work on a larger scale to see if similar themes are found across a more diverse group of people with PoTS. It will also be important to identify if there are differences across cultures, as well as across healthcare systems in different countries. Furthermore, we did not use a standardised measure of QoL in this study, future research would benefit from including this alongside the qualitative data, particularly when looking at differences across cultures and countries.

Finally, there will almost certainly have been an impact of COVID-19 on the data collection for this study. This is an extreme and unprecedented situation that was occurring alongside participants recording for this project. In some ways, the situation may have allowed participants more time to engage in the project, and the significance of the situation may have encouraged more self-reflection. However, it equally may have served as a distractor and made it more difficult to focus on the study. The recording took place as we were going into the first lockdown and there was significant uncertainty around risk status for this condition at the time, which may have contributed to participant’s experience of feeling misunderstood.

### Recommendations

1. This research suggests that further work educating HCPs, other community services and the wider population about this condition is urgently needed. HCPs must be able to identify symptoms of PoTS and refer patients for autonomic testing to enable early diagnosis. Educating these professional groups about this condition is also paramount to help participants feel validated. Ensuring HCPs understand the multitude of ways PoTS can impact the lives of their patients will remove the need for patients to feel they must defend themselves or their difficulties, particularly as the majority of the symptoms are not outwardly visible. Receiving diagnosis, recognition and empathy as soon as symptoms onset is a potentially potent and genuine therapy intervention.
2. Support for the carers and networks of people with this condition is key to help them better understand what people with PoTS are experiencing and how best to support them. This paper highlighted that experiencing the unpredictable loss of control and lack of agency over their body can be difficult to comprehend if not experienced. This can impact important relationships in a vicious cycle. Post-diagnosis sessions with key family or friends in the client’s support network to educate them on the condition would be beneficial, as well as access to support should family members struggle further down the line. The PoTS UK charity currently offers a support group to parents of young people with PoTS which they could consider widening to other carers and family members of all PoTS clients.
3. The importance of psychological support for this group has been reinforced by this study and ideally should be available from the point of diagnosis if needed, as the journey to diagnosis alone can be difficult. All participants discussed the impact on their mental well-being, many reporting feeling isolated, lonely and depressed at the time. This support needs to be from professionals aware of the unique challenges posed by the condition. The PoTS UK charity is launching a pilot scheme for geographical area support groups run by and for patients, with charity oversight, to tackle loneliness and isolation and support the emotional well-being of clients with PoTS.
4. From a practical point of view, participants spoke about the difficulties attending many medical appointments, often in different locations, and a lack of joined up care. Therefore, it may be beneficial for psychological support to be available in a flexible manner, ideally as part of a holistic PoTS clinic. Having sessions remotely so participants can attend from home may increase their ability to engage. To compliment more flexible psychological input, the findings from this study will also be informing an app that incorporates support around emotional well-being and acceptance, alongside lifestyle advice, which is currently in development.

### Future research

Future research into the efficacy of specific therapy approaches within this population will be beneficial to start building an evidence base around the most effective support. To address the limitations of this study, future research could expand recruitment outside of a purely digital space, which may increase the accessibility of studies and ensure a more representative population. Additionally, more research is needed to focus and refine treatments for, and understanding of, this condition. There is currently a lack of randomised controlled trials in this area, either on medication or which lifestyle factors are most effective with which groups. Although it is not life threatening, as demonstrated by this study and previous work in this area, PoTS significantly affects a person's QoL,^23,36,37^ so it is vital that further development of interventions informed by research of this type continue. As one participant noted ‘there‘s more to life than just staying alive’, and these findings suggest some key changes and strategies to enable lives well lived, and society contributed to optimally, by people with PoTS.
